# Parameters of pulse wave velocity: determinants and reference values assessed in the population-based study LIFE-Adult

**DOI:** 10.1007/s00392-018-1278-3

**Published:** 2018-05-15

**Authors:** Daniel Baier, Andrej Teren, Kerstin Wirkner, Markus Loeffler, Markus Scholz

**Affiliations:** 1 Institute for Medical Informatics, Statistics and Epidemiology, University of Leipzig, Haertelstrasse 16-18, 04107 Leipzig, Germany; 2 LIFE Research Center for Civilization Diseases, Leipzig, Germany; 3 Heart Center Leipzig, Leipzig, Germany

**Keywords:** Arterial stiffness, Blood pressure, Cardiovascular risk stratification, Brachial to ankle, Brachial to femoral, Carotid to femoral

## Abstract

**Aims and background:**

Parameters of arterial stiffness such as pulse wave velocity (PWV) were recently proposed as independent risk factors of cardiovascular events. We analyse three PWV parameters in the large population-based study LIFE-Adult to identify risk factors, normal and reference values.

**Methods and results:**

Brachial-ankle (ba), brachial-femoral (bf) and carotid-femoral (cf) PWV assessment was performed using Vicorder device. 8509 participants aged 19–80 were analysed. PWV parameters were moderately correlated (*r*(ba/bf) = 0.6, *r*(ba/cf) = 0.46, *r*(bf/cf) = 0.59). Age and blood pressure are the dominant determinants of PWV parameters explaining > 18% of variability. Sex was only relevant for bfPWV and cfPWV. All further analysed cardiovascular and other risk factors are of minor importance. We provide age-dependent percentiles for the population (reference values) and for the subgroup of normotonic individuals. All percentiles show a strong increase with age. The difference between normotonic and all individuals is small for younger age groups but increases up to 1 m/s for elderly subjects.

**Conclusion:**

Our study confirms and further underpins the strong impact of age and blood pressure on arterial stiffness and the relatively weak contribution of other factors, supporting an independent role of arterial stiffness in cardiovascular disease development. Age-dependent reference and normal values were provided on the basis of the so far largest study sample facilitating the implementation of PWV assessment in clinical practice. Due to better compliance, handling and stronger association with age and blood pressure, baPWV could serve as an alternative to cfPWV. Follow-up data are required to estimate the clinical significance of specified PWV cut-offs.

## Background

Cardiovascular disease (CVD) represents the leading cause of morbidity and mortality worldwide [1]. Among markers to assess CVD and its subclinical signs, arterial stiffness has proven to be an important predictor of cardiovascular (CV) events [2]. Arterial stiffness is a concept that refers to the material properties of the arterial wall, which in turn has functional consequences for the artery because it affects the manner in which pressure, blood flow and arterial diameter change with each heartbeat [3]. Within major arteries, stiffness has significant hemodynamic consequences such as reduced compliance and buffering capacity. This results in a premature wave reflection [4] and elevated systolic blood pressure (BP) [5]. Consequently, left ventricular afterload increases, which can lead to left ventricular hypertrophy and increased myocardial oxygen demand [6], whereas coronary perfusion decreases due to diastolic dysfunction [7, 8].

Different parameters of pulse wave velocity (PWV) are used to assess arterial stiffness, among others: brachial to ankle (baPWV), carotid to femoral (cfPWV) and brachial to femoral PWV (bfPWV). Currently, cfPWV is considered the gold standard because of its reliability [9–11]. A large body of evidence demonstrates its predictive value for CV events independent of traditional risk factors [2, 9, 12–18]. Devices used in these studies assessed cfPWV mostly by applanation tonometry or piezoelectric pressure transduction requiring highly experienced and trained operators. The more user—friendly oscillometric assessment of PWV implemented in Vicorder (Vicorder, Skidmore medical, UK) was shown to be comparable with the techniques mentioned above [19]. However, cfWV assessment as such presumes high degree of technical precision required for carotid and femoral pulse acquisition especially in obese subjects [20, 21]. In this regard, baPWV appears to be a good alternative. It can be easily obtained by wrapping blood pressure cuffs on the extremities which is more convenient for patients and more easily to apply for technical staff [21, 22].

As another option, bfPWV measurement focuses more on central components of the arterial tree, but avoids the uncomfortable neck cuff. Pulse wave acquisition of bfPWV has the advantage of showing no venous artefacts and the measurement is well tolerated even by children [20]. Assessment of bfPWV was rather of exploring interest, since this parameter has been only scarcely investigated so far.

While previous studies demonstrated that age and hypertension [14, 23, 24] strongly influence PWV, the impact of other conventional atherosclerotic risk factors, such as diabetes mellitus [25], metabolic syndrome [26–28], and smoking [29, 30] is inconsistent. Reference values based on a large-scale European population have only been established for the gold standard cfPWV by one multi-centre study [14]. In spite of its growing importance, to our knowledge, reliable reference values for baPWV, only exist for Asian populations [31, 32]. Similarly, no reference data are available for bfPWV so far.

In the present paper, we used data from the LIFE-Adult study, a large population-based study conducted in the city of Leipzig, Germany, to analyse and compare the three different parameters of pulse wave velocity cfPWV, baPWV and bfPWV, all measured by Vicorder device. Additionally, we examined the influence of CV risk factors on PWV. Provision of normal and reference values in dependence on age for each PWV parameter was another major objective of our study.

## Methods

### Study population

Analyses were performed in the LIFE-Adult study, a single centre, population-based cohort study conducted by the Leipzig Research Centre for Civilization Disease (LIFE). Its objective is to investigate prevalence and early onset markers of civilization diseases, with a major focus on atherosclerosis and vascular disease. Between August 2011 and November 2014 a total of 10,000 participants, with ages ranging from 18 to 80 years underwent an extensive assessment programme including structured interviews, questionnaires, physical and instrumental examinations and biospecimen collection. Details of the study can be found elsewhere [33].

Arterial stiffness was determined by pulse wave velocity and pulse wave analysis. In the present analysis, only participants with at least one brachial-ankle or one carotid-femoral PWV measurement were included, resulting in a total sample size of 8509 subjects. Brachial-femoral PWV assessment was available for 3904 of these individuals (see below).

To determine reference values we analysed all individuals. To determine normal values, we analysed a subgroup of 3099 normotonic participants. Probands were included in this subgroup when blood pressure was optimal or normal (systolic BP < 130 mmHg/diastolic BP < 85 mmHg) according to the ESH/ESC guidelines [11] and their medical history revealed neither diagnosis nor medication of hypertension nor prevalent CVD.

### Pulse wave measurement

All PWV measurements were performed as previously described, using the oscillometry-based Vicorder device (Vicorder, Skidmore medical, Bristol, UK) [10]. Validity, intra- and inter-rater reliability and repeatability of the Vicorder device were shown to be good (including in our own hands) [10, 19, 34].

PWV was determined as the ratio of pulse travel distance to pulse transit time (PTT) derived from 2-point diastolic pulse wave analysis. PTT was determined from foot-to-foot real time shift between simultaneous 2-point-recorded pulse wave curves using an in-built cross-correlation algorithm based on the peak of the second derivative of the pressure curve. Pulse waves were recorded upon automatic cuff inflation to approximately 60 mmHg over at least 10 pulsations. Furthermore, central systolic and diastolic blood pressure and augmentation index were assessed applying device-specific brachial pulse wave analysis. All travel distances were measured separately for each assessment using a flexible tape. In case of significant android fat distribution, a slide caliper was used [10]. Travel distance for cfPWV was measured directly from the suprasternal notch to the center of the femoral cuff. BaPWV and bfPWV were measured directly from the center of the brachial to the center of the ankle and femoral cuff, respectively (see Fig. 2 in [10]).

Data regarding acceptability and global time requirements for the Vicorder devices were collected and published elsewhere in detail [35]. All PWV modes were measured subsequently on the right side of the body. For the first half of the study, two measurements of both, baPWV and cfPWV, were performed. Since correlations between the first and second value were high, we used the first value for all analyses to be consistent with the second half of the study. bfPWV was available for a total of 3968 participants and two measurements were performed for most of them (*n* = 3954). Since variance of bfPWV was significantly higher than those of baPWV and cfPWV, we decided to average these two values. Measurements are discarded if the difference of first and second measurement is > 5 m/s (baPWV), > 10 m/s (cfPWV) and > 25 m/s (bfPWV), respectively. This choice is based on a practical, data driven approach resulting in roughly homogeneous distributions of first and second measurements around the diagonal (data not shown). Resulting intra-individual reliabilities assessed in terms of the concordance correlation coefficient (CCC) are high (baPWV: CCC = 0.94, cfPWV: CCC = 0.80, bfPWV: CCC = 0.84) and are in excellent agreement to those observed in our former study [10].

In summary, the following sample sizes for the different PWV parameters are available: 8483 for baPWV, 8460 for cfPWV and 3904 for bfPWV. Obesity status is a strong predictor for an unsuccessful PWV assessment. For example, regarding cfPWV, assessment was unsuccessful in 2.3% of non-obese but in 10.4% of obese subjects (odds ratio = 4.9, *p* < 0.001).

### Laboratory measurements

In the LIFE-Adult study, an extensive panel of laboratory tests covering 83 analytes and biomarkers was performed on fresh biospecimen directly on the day of sample collection in a highly standardized manner [33]. In the present paper, we analysed plasma or serum levels of the following parameters for correlation with PWV parameters: haemoglobin A1c (HbA1c), fasting plasma glucose (FPG), fasting insulin (INS), uric acid (UA), cardiac-specific Troponin T (TNT), pro brain natriuretic peptide (PBNP), total cholesterol (TC), high-density lipoprotein cholesterol (HDL), low-density lipoprotein cholesterol (LDL), triglycerides (TG), lipoprotein A (LPA), cystatin C (CSYC), creatinine (CR), urea (UR) and high sensitivity C-reactive protein (hsCRP). All blood samples were obtained in a fasting state of at least 8 h. Due to incomplete measurements of some laboratory parameters, numbers may slightly deviate from 8509 for some analytes.

### Other assessments

Blood pressure was measured three times at 3-min intervals using an automatic oscillometric blood pressure monitor (OMRON 705IT, OMRON Medizintechnik Handelsgesellschaft mbH, Mannheim, Germany) after resting for at least 5 min. The first measurement was discarded and the second and third measurements were averaged to obtain the blood pressure.

All participants provided a medical history and underwent anthropometric measurements. Prevalent diabetes was determined by fulfilling at least one of the following conditions: HbA1c > 6.5%, the use of hypoglycaemic medications or self-reported diabetes.

Medication was categorized according to ATC code lists. For anti-hypertensive drugs, we summarized the codes C02, C03, C07, C08, and C09 as recommended [36].

### Statistical analysis

Data are expressed as mean (± SD) or median (interquartile range) for continuous variables or number (proportion) of participants for categorical variables. Statistical analysis was performed using IBM SPSS Statistics, version 24.0 (Armonk, NY: IBM Corp.). All PWV modes, the laboratory parameters as well as weight and body mass index (BMI) were logarithmically transformed to approximate normal distributions.

Differences between two groups were compared by the independent samples *t* test. Linear correlations were determined using Pearson’s *r* correlation coefficient and linear regression analysis was performed to evaluate the association between each PWV parameter and other clinical covariates. Multiple regression analysis was performed using a stepwise regression approach. In each step, a variable is considered for addition to (*p* value of *F* Test < 0.05) or subtraction from (*p* value of *F* Test > 0.10) the set of explanatory variables (forward–backward model selection as implemented in SPSS). This method was chosen to identify independent factors determining PWV. To avoid collinearity issues we only considered SBP as representative for blood pressure for this kind of analysis.

Means (± 2SD) and medians (95%-interval) of each PWV parameter were calculated according to age category for the normotonic subpopulation (see above) and for the entire study population, to deduce normal and reference ranges of PWV. We also applied quantile regression to obtain smooth reference curves. The *gamlss* package of the statistics software *R* (http://www.r-project.org) was used for that purpose. Since bf-mode showed the highest variance with less than half of the measurements available for the modes cf and ba, quantile regression was stabilized by winsorizing values above 60 m/s. 11 such outliers were removed.

## Results

### Cohort description

Mean age of participants was 57.3 ± 12.4 years (range: 19–80), and 51.3% (*n* = 4365) of the subjects were women. Anthropometric, hemodynamic and laboratory data of all 8509 participants are shown in Table 1.

**Table 1:** Study description

	Mean ± SD	Median (interquartile range)
Age (years)	57.3 ± 12.4	57.7 (47.6–68.0)
Male/Female (%)	4145/4364 (48.7/51.3)	
Height (m)	1.71 ± 0.24	1.70 (1.63–1.77)
Weight (kg)	79.0 ± 28.8	77.0 (67.3–87.6)
BMI (kg/m²)	27.0 ± 4.5	26.5 (23.8–29.7)
WHR	0.9 ± 0.1	0.93 (0.86–0.99)
Waist (cm)	96 ± 13	95 (87–104)
SBP (mmHg)	128 ± 17	127 (117–138)
DBP (mmHg)	75 ± 10	75 (69–82)
cSBP (mmHg)	130 ± 16	129 (120–140)
cDBP (mmHg)	75 ± 9	74 (68–81)
AI (%)	20 ± 7	21 (16–25)
HR (bpm)	70 ± 11	69 (63–77)
ABI	1.1 ± 0.1	1.1 (1.0–1.2)
Diabetes controls/cases (%)	7528/953 (88.8/11.2)	
FPG (mmol/l)	5.7 ± 1.1	5.4 (5.0–5.9)
INS (pmol/l)	65.6 ± 97.1	51.9 (35.7–77.8)
UA (µmol/l)	321.3 ± 83.5	316.0 (260.0–376.0)
TC (mmol/l)	5.6 ± 1.1	5.5 (4.9–6.3)
HDL (mmol/l)	1.6 ± 0.5	1.6 (1.3–1.9)
LDL (mmol/l)	3.5 ± 1.0	3.4 (2.8–4.1)
TG (mmol/l)	1.4 ± 1.0	1.2 (0.8–1.6)
LPA (g/l)	0.2 ± 0.3	0.10 (0.04–0.27)
TNT (pg/ml)	6.2 ± 5.7	4.7 (3.0–7.2)
PBNP (pg/ml)	114.3 ± 231.8	63.9 (34.8–117.6)
CYSC (mg/l)	0.9 ± 0.2	0.9 (0.8–1.0)
CR (µmol)	80.0 ± 16.8	78.0 (69.0–89.0)
Urea (mmol/l)	5.0 ± 1.5	4.8 (4.0–5.7)
eGFR (ml/min/1.73m²)	84.9 ± 19.1	83.8 (72.1–96.8)
hsCRP (mg/l)	2.7 ± 5.2	1.5 (0.8–2.9)
Smoking Never/Ex/Current (%)	3956/2398/1814 (48.4/29.4/22.2)	
Anti-diabetic Drugs yes/no (%)	730/7764 (8.6/91.4)	
Anti-hypertensive Drugs yes/no (%)	3527/4967 (41.5/58.5)	
Drugs influencing lipid metabolism yes/no (%)	1073/7421 (12.6/87.4)	

Description of anthropometric, hemodynamic and laboratory data of all subjects (*n* = 8509). Means and standard deviations as well as medians and interquartile ranges are presented

*BMI* body mass index, *WHR* waist-to-hip ratio, *SBP* systolic blood pressure, *DBP* diastolic blood pressure, *cSBP* central systolic blood pressure, *cDBP* central diastolic blood pressure, *AI* augmentation index, *HR* heart rate, *ABI* ankle brachial index, *FPG* fasting plasma glucose, *INS* fasting insulin, *UA* uric acid, *TC* total cholesterol, *LDL* low-density lipoprotein cholesterol, *HDL* high-density lipoprotein cholesterol, *TG* triglyceride, *LPA* lipoprotein A, *TNT* cardiac-specific Troponin T, *PBNP* pro brain natriuretic peptide, *CYSC* cystatin C, *CR* creatinine, *UR* urea, *eGFR* estimated glomerular filtration rate, *hsCRP* high sensitivity C-reactive protein

**Fig. 1:**
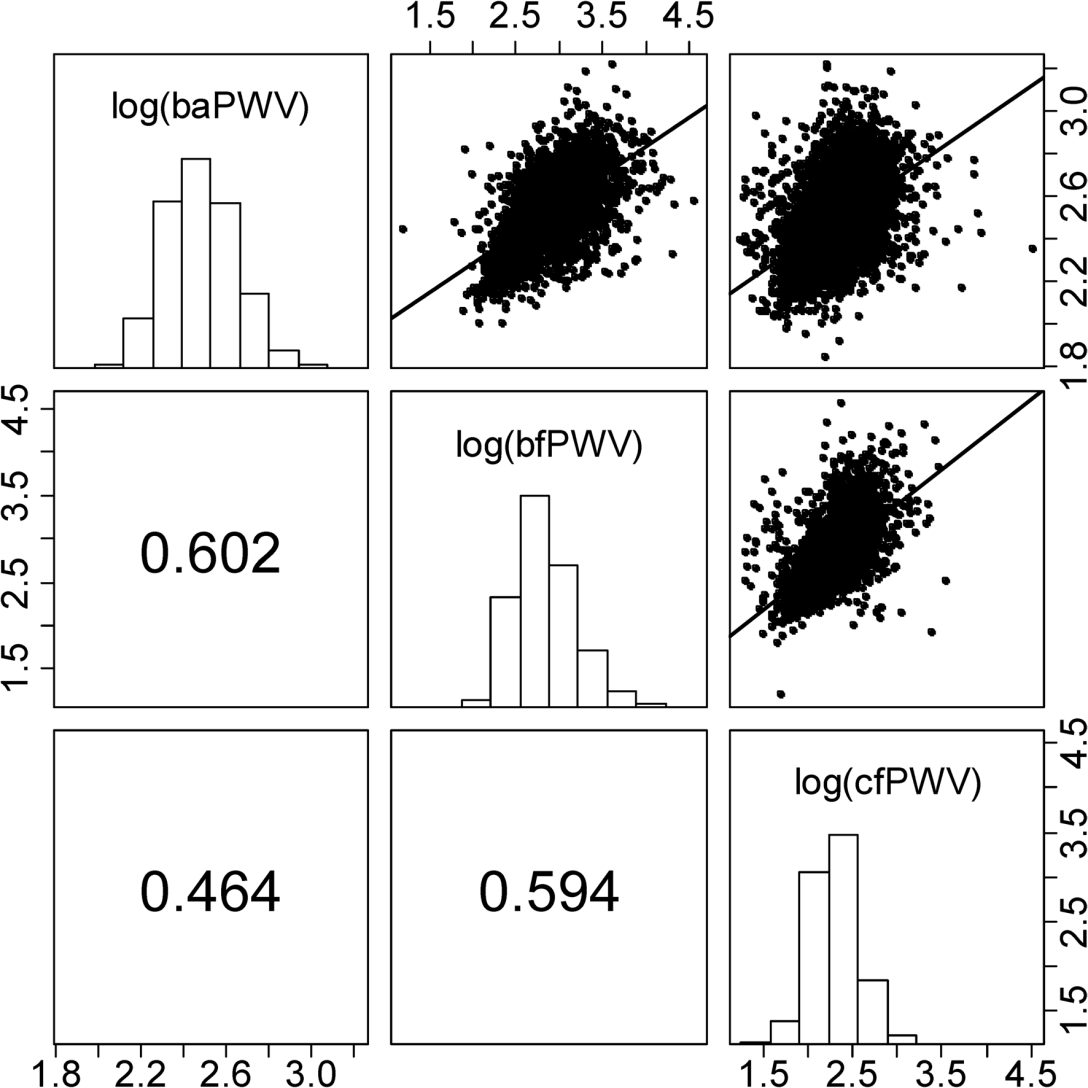
(Distribution and correlation of the three PWV parameters): We present histograms of logarithmized PWV parameters baPWV, bfPWV and cfPWV in the diagonal, Pearson’s correlations in the lower triangle and scatterplots and linear regression lines in the upper triangle of the matrix

Figure 1 depicts the distribution and correlation of the three PWV parameters. Among the PWV parameters, bfPWV showed the largest variation (variance coefficient (VC): 12.6%) while baPWV showed the smallest variation (VC: 6.8%). Of note, pulse wave parameters are only moderately correlated with the strongest correlation observed between baPWV and bfPWV (*r* = 0.60).

### Univariate association analysis

Linear regression analysis between PWVs and other clinical variables showed that anthropometric, hemodynamic, cardiac, renal and metabolic variables, atherosclerotic risk factors and medications were univariate associated with all PWV parameters (Table 2). Only Ankle-Brachial-Index and Lipoprotein A did not correlate with any of the PWV parameters. Also, associations for bfPWV and cfPWV are generally weaker than those for baPWV, especially regarding lipid status (e.g., triglycerides *r* = 0.25 for baPWV compared to *r* = 0.12 and *r* = 0.14 for bfPWV and cfPWV respectively). Remarkably, in contrast to common expectation, smoking status correlated negatively with both, baPWV and bfPWV.

**Table 2:** Pearson coefficients of correlation for linear regression analysis between PWV parameters and other clinical variables

	baPWV	bfPWV	cfPWV
*N*	8483	3904	8460
Covariate	Pearson’s* r*	Pearson’s *r*	Pearson’s *r*
*Demography*			
Age	0.66	0.48	0.39
Age group	0.64	0.47	0.15
Sex	− 0.17	0.12	0.05*
*Anthropometry*			
Height	− 0.09	− 0.21	− 0.12
Weight	0.16	0.069*	0.10
BMI	0.24	0.23	0.21
WHR	0.41	0.13	0.15
Waist	0.34	0.21	0.21
*Hemodynamic parameters*			
BP-Cat	0.48	0.22	0.27
SBP	0.50	0.25	0.29
DBP	0.25	− 0.018*	0.16
cSBP	0.53	0.26	0.37
cDBP	0.32	− 0.017*	0.20
AI	0.26	0.20	0.23
HR	0.11	0.062*	0.062*
ABI	0.036*	0.003*	0.002*
*Metabolic parameters*			
Diabetes	0.24	0.19	0.13
FPG	0.38	0.23	0.19
INS	0.25	0.16	0.15
UA	0.29	0.11	0.093*
Lipid status			
TC	0.15	0.088*	0.13
LDL	0.15	0.072*	0.11
HDL	− 0.11	− 0.012*	− 0.007*
TG	0.25	0.12	0.14
LPA	0.025*	0.036*	0.028*
Cardiac parameters			
TNT	0.39	0.24	0.18
PBNP	0.27	0.26	0.18
Renal parameters			
CYSC	0.37	0.27	0.20
CR	0.16	0.035*	0.015*
UR	0.21	0.13	0.088*
eGFR	− 0.43	− 0.32	− 0.24
Inflammation parameter			
hsCRP	0.17	0.18	0.15
Risk behaviour			
Smoking	− 0.12	− 0.12	− 0.06*
Medication			
Anti-diabetic drugs	0.21	0.18	0.12
Anti-hypertensive drugs	0.36	0.28	0.20
Drugs influencing lipid metabolism	0.21	0.15	0.11

All PWV and laboratory parameters, weight and BMI were logarithmized prior to analysis (in order to approximate normal distributions). Only variables with *r* > 0.1 were considered to be correlated

*BMI* body mass index, *WHR* waist-to-hip ratio, *BP-Cat*. blood pressure category, *SBP* systolic blood pressure, *DBP* diastolic blood pressure, *cSBP* central systolic blood pressure, *cDBP* central diastolic blood pressure, *AI* augmentation index, *HR* heart rate, *ABI* ankle brachial index, *FPG* fasting plasma glucose, *INS* fasting insulin, *UA* uric acid, *TC* total cholesterol, *LDL* low-density lipoprotein cholesterol, *HDL* high-density lipoprotein cholesterol, *TG* triglyceride, *LPA* lipoprotein A, *TNT* cardiac-specific Troponin T, *PBNP* pro brain natriuretic peptide, *CYSC* cystatin C, *CR* creatinine, *UR* urea, *eGFR* estimated glomerular filtration rate, *hsCRP* high sensitivity C-reactive protein

*No significant correlation

### Multivariate association analysis

The results of stepwise multiple regression analysis revealed several independent variables associated with PWV parameters. Age is by far the strongest independent contributor to PWV variability (partial ϱ ranging from 0.27 for cfPWV to 0.44 for baPWV) followed by systolic blood pressure (partial ϱ ranging from 0.15 for bfPWV to 0.28 for cfPWV, Table 3), except for bfPWV where sex is the second strongest independent contributor (*ϱ* = 0.23).

**Table 3:** Results of multiple regression analysis of the different PWV parameters with CV risk factors and other clinical variables

Covariate	*B*	Beta	*T*	*p* value	95%-CI (B)		*ρ*	cum. R^2^
*Multiple regression analysis of baPWV*								
Constant	1.60		38.3	< 0.001	1.52	1.69		
**Age**	0.0069	0.51	55.5	< 0.001	0.0066	0.0071	0.439	0.442
**SBP**	0.0031	0.30	34.9	< 0.001	0.0029	0.0033	0.276	0.547
**WHR**	0.20	0.11	9.54	< 0.001	0.16	0.24	0.076	0.563
TG	0.021	0.064	6.84	< 0.001	0.015	0.027	0.054	0.568
FPG	0.049	0.048	4.28	< 0.001	0.027	0.072	0.034	0.571
Smoke	− 0.0075	− 0.035	− 4.27	< 0.001	− 0.011	− 0.0041	− 0.034	0.572
hsCRP	0.0079	0.044	5.12	< 0.001	0.0049	0.011	0.040	0.572
BMI	− 0.092	− 0.087	− 7.93	< 0.001	− 0.11	− 0.069	− 0.063	0.575
INS	0.010	0.035	3.24	0.001	0.0038	0.016	0.026	0.576
Diabetes	0.017	0.031	3.16	0.002	0.0065	0.028	0.025	0.577
UA	0.013	0.021	2.16	0.031	0.0012	0.026	0.017	0.577
*Multiple regression analysis of bfPWV*								
Constant	0.33		1.71	0.088	− 0.049	0.71		
**Age**	0.011	0.39	20.6	< 0.001	0.010	0.012	0.390	0.216
**Sex**	0.17	0.23	11.4	< 0.001	0.14	0.20	0.229	0.244
**SBP**	0.0030	0.14	7.49	< 0.001	0.0022	0.0037	0.152	0.264
**BMI**	0.23	0.099	5.07	< 0.001	0.14	0.32	0.104	0.280
Diabetes	0.076	0.068	3.72	< 0.001	0.036	0.12	0.076	0.284
UA	0.078	0.057	2.55	0.011	0.018	0.14	0.052	0.286
*Multiple regression analysis of cfPWV*								
Constant	0.72		7.68	< 0.001	0.54	0.91		
**Age**	0.0079	0.36	25.1	< 0.001	0.0073	0.0085	0.271	0.155
**SBP**	0.0035	0.20	17.5	< 0.001	0.0031	0.0038	0.189	0.188
**Sex**	0.078	0.14	10.2	< 0.001	0.063	0.093	0.110	0.199
**Weight**	0.14	0.10	7.30	< 0.001	0.11	0.18	0.079	0.209
TG	0.024	0.045	3.77	< 0.001	0.011	0.036	0.041	0.211
TNT	− 0.028	− 0.053	− 3.63	< 0.001	− 0.043	− 0.013	− 0.039	0.213
hsCRP	0.0071	0.025	2.12	0.034	0.00053	0.014	0.023	0.213
Drug hyper	− 0.017	− 0.030	− 2.32	0.020	− 0.031	− 0.0026	− 0.025	0.214
Drug diab	0.027	0.026	2.28	0.023	0.0037	0.049	0.025	0.214

All PWV and laboratory parameters, weight and BMI were logarithmized prior to analysis. For the final model, we present name of covariate ranked by model selection, regression coefficient (*B*), standardized regression coefficient (Beta), *T* statistics (*T*), *p* value, 95% confidence interval of *B*, partial correlation (ρ) and cumulative explained variance *R*^2^. Relevant covariables which explain at least 1% additional variance were depicted in bold. Units are the same as in Table 1

*SBP* systolic blood pressure, *WHR* waist-to-hip ratio, *TG* triglyceride, *BMI* body mass index, *FPG* fasting plasma glucose, *INS* fasting insulin, *hsCRP* high sensitivity C-reactive protein, *UA* uric acid, *TNT* cardiac-specific Troponin T, *Drug hyper* anti-hypertensive drugs, *Drug diab* anti-diabetic drugs

Due to our large cohort, many variables are significant in the final regression models but do not contribute relevantly in terms of explained variance. Restricting the models to variables which account for at least additional 1% of the explained variance, only three (baPWV), respectively, four (bfPWV, cfPWV) parameters contribute relevantly (Table 3). Besides age and systolic blood pressure, these comprise sex (bfPWV, cfPWV) and the adiposity related measures WHR (baPWV), BMI (bfPWV) and weight (cfPWV).

### Age dependence and reference values

**Fig. 2:**
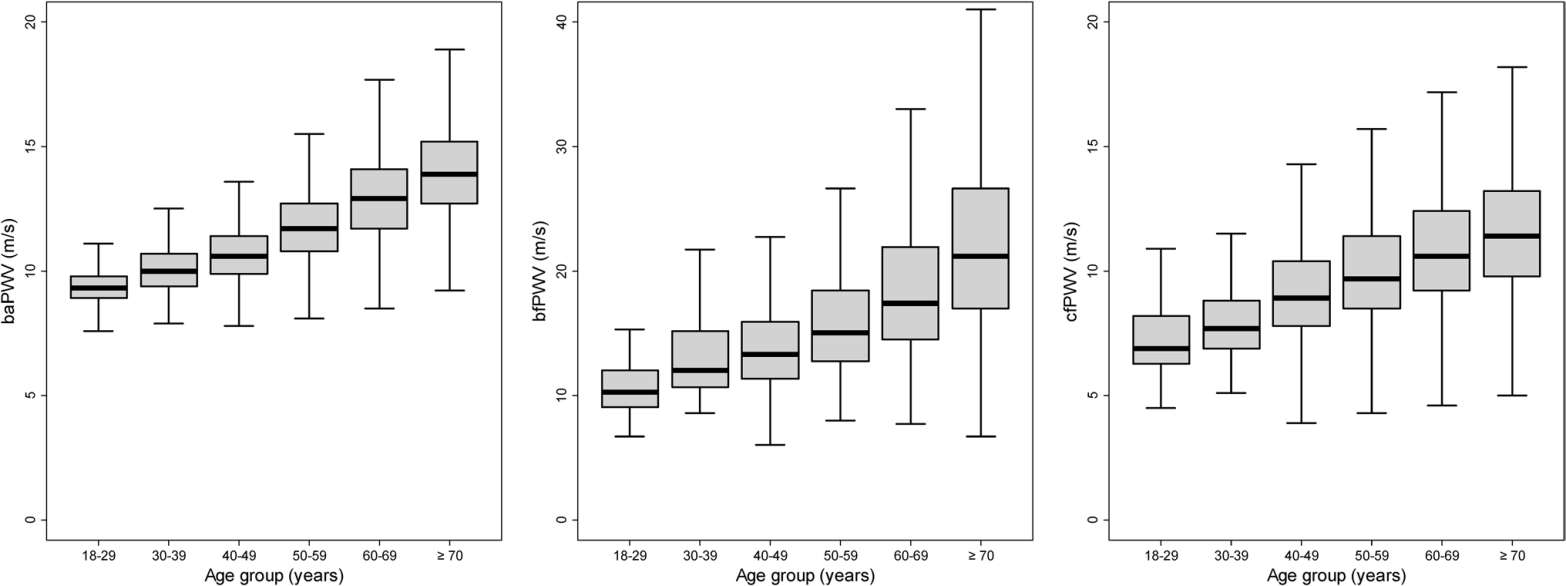
(Age dependence of PWV parameters): We present box plots in dependence on age groups for all PWV parameters. Outliers are not displayed. A clear increasing trend with age can be detected. Note that a larger scale was used for bfPWV for better readability

All PWV parameters continuously increase with age. We arranged all ages in groups of decades and present box plots of PWV parameters (Fig. 2).

To obtain normal and reference values for the three different PWV parameters, we calculated means ± 2SD and medians with the 2.5 and 97.5th percentile for each age category in (1) the whole study population (SBP mean ± SD: 128 ± 17 mmHg, DBP mean ± SD: 75 ± 9.9 mmHg) and (2) the subgroup of normotonic subjects (SBP mean ± SD: 116 ± 8.6 mmHg, DBP mean ± SD: 71 + 6.8 mmHg). Results are shown in Table 4. Across the age categories, mean normal values rise from 9.2 (age category 18–30 years) to 13.0 m/s (age category 70–80 years) for baPWV, from 10.4 to 19.4 m/s for bfPWV and from 7.2 to 10.5 m/s for cfPWV. For mean reference values, these ranges are 9.3–13.9 m/s, 10.7–21.7 m/s and 7.3–11.4 m/s, respectively.

**Table 4:** Normal and reference values of PWV parameters

baPWV	Normal values (*n* = 3099)		Reference values (*n* = 8483)	
Age category (years)	Mean (± 2SD)	Median (ref. range)	Mean (± 2SD)	Median (ref. range)
18–29	9.2 (7.9–10.8)	9.2 (7.8–11.1)	9.3 (7.9–11.0)	9.3 (7.8–11.4)
30–39	9.9 (8.2–11.8)	9.8 (8.2–11.8)	10.0 (8.2–12.2)	10.0 (8.3–12.3)
40–49	10.3 (8.5–12.5)	10.2 (8.6–12.5)	10.7 (8.5–13.4)	10.6 (8.7–13.7)
50–59	11.1 (8.8–13.8)	11.0 (9.0–13.6)	11.8 (8.9–15.5)	11.7 (9.2–15.9)
60–69	12.1 (9.4–15.5)	12.0 (9.7–15.9)	12.9 (9.8–16.9)	12.9 (10.1–17.1)
≥ 70	13.0 (10.1–16.9)	13.0 (10.3–17.2)	13.9 (10.6–18.4)	13.9 (10.6–18.4)

bfPWV	Normal values (*n* = 1168)		Reference values (*n* = 3904)	
Age category (years)	Mean (± 2SD)	Median (ref. range)	Mean (± 2SD)	Median (ref. range)
18–29	10.4 (6.1–17.9)	9.9 (6.8–24.4)	10.7 (6.4–17.7)	10.3 (6.9–21.9)
30–39	12.9 (6.8–24.4)	12.1 (8.8–38.4)	13.0 (7.1–23.9)	12.1 (9.0–28.1)
40–49	14.0 (7.4–26.3)	13.2 (9.3–31.1)	14.0 (7.8–25.2)	13.3 (9.4–28.3)
50–59	14.9 (7.9–28.3)	14.2 (9.3–34.2)	15.8 (8.5–29.5)	15.1 (9.9–33.5)
60–69	16.7 (8.9–31.1)	15.9 (10.6–36.3)	18.1 (9.8–33.4)	17.4 (11.0–36.8)
≥ 70	19.2 (9.4–38.9)	18.9 (10.6–41.2)	21.7 (10.9–43.2)	21.2 (12.0–47.4)

cfPWV	Normal values (*n* = 3092)		Reference values (*n* = 8460)	
Age category (years)	Mean (± 2SD)	Median (ref. range)	Mean (± 2SD)	Median (ref. range)
18–29	7.2 (4.2–12.4)	6.8 (5.0–13.1)	7.3 (4.3–12.5)	6.9 (5.1–13.1)
30–39	7.8 (5.0–12.2)	7.6 (5.5–14.0)	7.9 (5.0–12.4)	7.7 (5.7–13.9)
40–49	8.9 (5.4–14.5)	8.6 (6.0–15.2)	9.1 (5.6–14.8)	8.9 (6.0–15.7)
50–59	9.4 (5.9–15.1)	9.2 (6.1–16.1)	9.9 (6.0–16.4)	9.7 (6.4–17.2)
60–69	10.1 (6.1–16.7)	9.9 (6.4–18.8)	10.7 (6.5–17.6)	10.6 (6.7–17.9)
≥ 70	10.5 (6.3–17.7)	10.6 (6.3–18.1)	11.4 (6.8–19.3)	11.4 (6.9–19.6)

We present means, standard deviations, medians and reference ranges (2.5–97.5th percentile) of the three PWV parameters (measured in m/s) according to age groups. We analysed all individuals (reference values) and the normotonic subgroup (normal values)

**Fig. 3:**
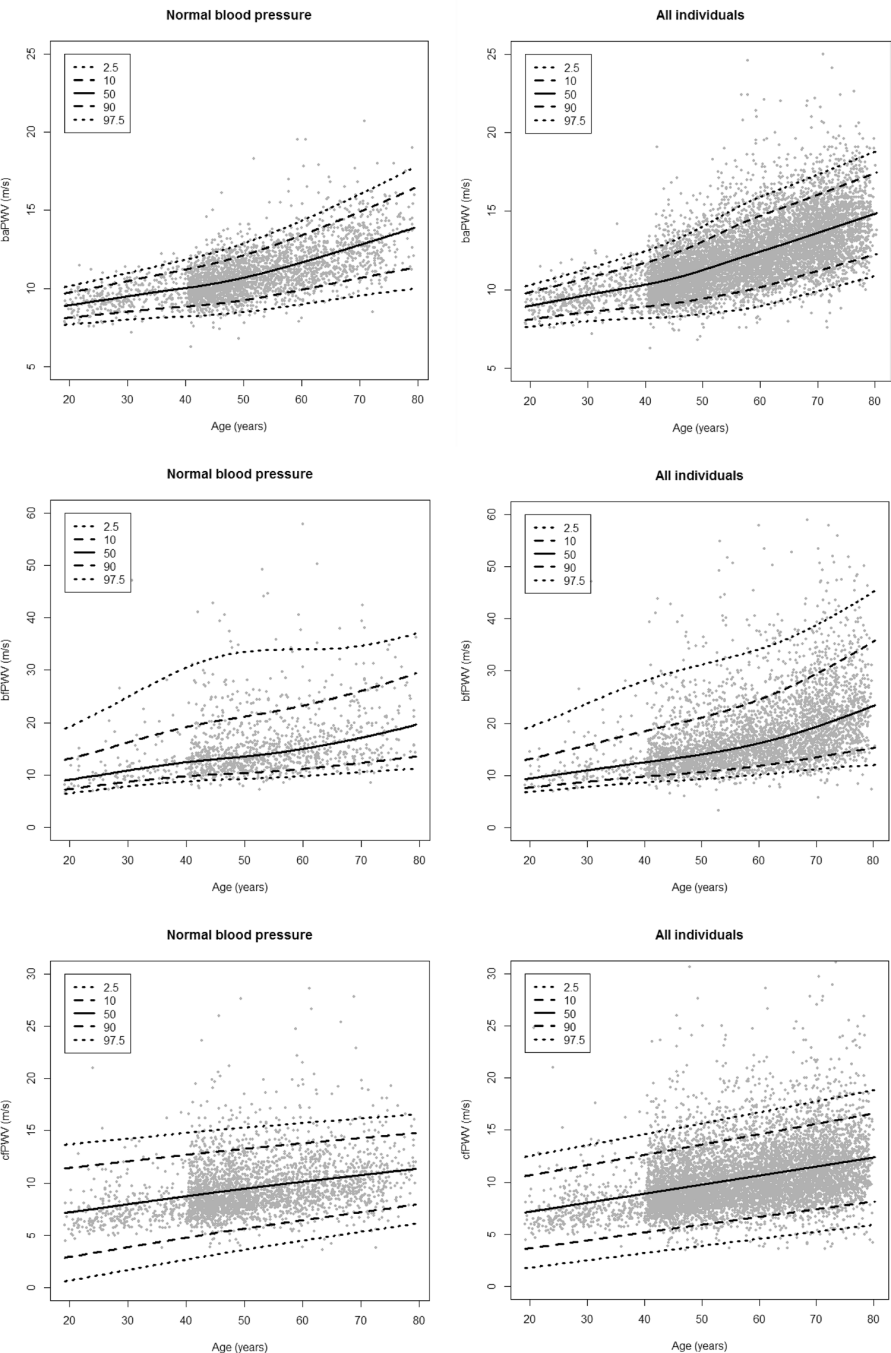
(Age-dependent percentile curves for PWV parameters): We present age-dependent percentile curves (2.5, 10, 50, 90 and 97.5th percentile) for all individuals (reference group) and for the subgroup of normotonic individuals

Age-dependent percentile curves for normal and reference values of PWV parameters are displayed in Fig. 3. Both groups show the same trends. While the reference group and the subgroup of normotonic individuals are almost at the same level for lower age groups, the differences between percentile curves increase up to about 1 m/s for the elderly.

## Discussion

Cardiovascular disease as the leading cause of mortality and morbidity worldwide is predicted to continue to increase in the coming years. Therefore, easy to handle, economical and widely applicable non-invasive assessments of early vascular damage are of great clinical significance. The objective of the present study was the evaluation of the three different PWV parameters baPWV, bfPWV and cfPWV in the large epidemiologic setting of the LIFE-Adult study. We compared these parameters, analysed corresponding influencing factors and established normal and reference values.

Although cfPWV is considered the gold-standard, baPWV has recently emerged to be a more feasible procedure promising better patient compliance. During our examinations we experienced that cfPWV assessment was sometimes hampered due to intolerance towards the neck cuff or in case of obesity, where the cuff did not fit around the neck or the upper thigh.

Strong positive correlations between cfPWV and baPWV [37] as well as between cfPWV and bfPWV [20] were reported, suggesting that all three parameters of PWV represent central arterial stiffness. In our study, we observed only moderate correlations between these parameters. This could be explained by methodological differences of tonometric and oscillometric devices in the detection of the pulse wave and by different methods for measuring the arterial path length. We confirmed the systematic shift of baPWV values compared to cfPWV values (baPWV being 17% higher in our data compared to 18% as observed in [37]).

In line with several previous reports, we observed that age and blood pressure are by far the most important factors influencing PWV parameters, explaining large parts of the overall variance [14, 16, 23, 24, 27, 31, 38]. Concerning the impact of other CV risk factors on PWV, literature is currently inconsistent. Besides age and blood pressure, BMI, central obesity, triglycerides (TG), high-density lipoprotein cholesterol (HDL), fasting plasma glucose (FPG) and uric acid (UA) were reported to be associated with baPWV [23, 26–28, 31]. We could confirm these significant associations in our cohort. However, these factors contribute only weakly in multivariate analysis. Concerning influencing factors for cfPWV, a review article summarized that the majority of studies found no independent association between cfPWV and sex, total cholesterol, LDL, HDL, TG, smoking or BMI [24]. In our study, none of these factors independently explained more than 1.2% of the variance of cfPWV explaining this observation.

Though smoking is strongly associated with peripheral vascular disease and arteriosclerosis, the impact on arterial stiffness has been reported inconsistently so far. Jatoi et al. showed that smoking status correlates positively and duration of smoking cessation negatively with cfPWV [30]. However, in a recent study, no association between smoking status and central arterial stiffness (cfPWV) was observed [39]. Only peripheral arterial stiffness (femoral to ankle PWV) showed weak negative correlation with smoking status. The authors concluded that the profound and well-documented adverse effects of cigarette smoking on the vasculature may not include a sustained stiffening of the arteries measured at older age. We could confirm this finding by observing an independent, but small negative correlation of smoking with baPWV, but not for the other PWV parameters.

Given the large evidence of PWV in predicting CV events but lack of strong dependence of PWV parameters on CV risk factors other than age and hypertension, we hypothesize that the prognostic value of PWV may be related to a process of arterial ageing or life time exposure of increased blood pressure independent of traditional CV risk factors [24]. In this sense, our study supports the recommendation of the ESH/ESC guidelines to implement PWV measurement in routine CV risk assessment, in addition to known atherosclerotic risk factors [11].

In these guidelines, a fixed threshold of 10 m/s was proposed for cfPWV regardless of age [11, 40]. However, this leads to a major part of the elderly population being classified at higher risk. For example, in our cohort, 64% of the probands with age > 60 years and even 51% of the normotonic subjects with age > 60 years have cfPWV > 10 m/s. Therefore, we provided age-dependent percentile curves for Vicorder-derived PWV parameters considered in the present study. Compared to a previous study on cfPWV in a large European population, our normal values are in good agreement. On average they are about 15–20% higher, except for the two highest age decades, which are approximately at the same level (i.e., 2–4% higher) [14]. This corresponds well to the standardization to direct path length and the rescaling factor of 0.8 used for cfPWV in the mentioned study [14].

Regarding baPWV, only reference values in an Asian cohort are available so far [31]. Compared to these data, our reference values of baPWV are about 50% lower. However, comparability is limited due to different techniques of pulse wave measurement (plethysmography vs. oscillometry) and determination of arterial path length (direct vs. estimated from body height). In addition, our study provides for the first time normal and reference values for bfPWV.

Several limitations of the present study are of note. The age group < 40 years is underrepresented in our study and corresponding PWV percentiles are, therefore, estimated with lower accuracy. The same holds for bfPWV for which only 3904 measurements are available in total. Again, follow-up data regarding future CV events are required to provide reasonable cut-offs of PWV parameters for intervention. Moreover, PWV assessment is significantly more difficult in obese subjects resulting in higher percentages of missing values. This especially applies for cfPWV.

In conclusion, we performed the so far largest population-based study of parallel assessment of three PWV parameters. We confirmed that age and blood pressure are the main determinants of PWV and showed that all other factors are of minor importance. We estimated normal and reference values for the three different PWV parameters in dependence on age facilitating the implementation of PWV assessments in ongoing and future clinical practice to improve cardiovascular risk stratification. Since baPWV reveals better handling and stronger association with age and blood pressure, it could serve as an alternative to cfPWV.

## Abbreviations

baPWV: Brachial to ankle pulse wave velocity
bfPWV: Brachial to femoral pulse wave velocity
BMI: Body mass index
cfPWV: Carotid to femoral pulse wave velocity
CR: Creatinine
CV: Cardiovascular
CVD: Cardiovascular disease
CYSC: Cystatin C
FPG: Fasting plasma glucose
HbA1c: Haemoglobin A1c
HDL: High-density lipoprotein cholesterol
hsCRP: High sensitivity C-reactive protein
INS: Fasting insulin
LDL: Low-density lipoprotein cholesterol
LPA: Lipoprotein A
PBNP: Pro brain natriuretic peptide
PTT: Pulse transit time
PWV: Pulse wave velocity
TC: Total cholesterol
TNT: Cardiac-specific Troponin T
TG: Triglycerides
UA: Uric acid
UR: Urea
VC: Variance coefficient
WHR: Waist-to-hip ratio
